# Evolving data analysis of an Oral Lipid Tolerance Test toward the standard for the Oral Glucose Tolerance Test: Cross species modeling effects of AZD7687 on plasma triacylglycerol

**DOI:** 10.1002/prp2.465

**Published:** 2019-03-09

**Authors:** Pablo Morentin Gutierrez, James Yates, Catarina Nilsson, Sue Birtles

**Affiliations:** ^1^ AstraZeneca R&D IMED DMPK Chesterford Science Park UK; ^2^ AstraZeneca R&D IMED ECD Gothenburg Sweden; ^3^ VWR Alderley Park UK

**Keywords:** AZD7687, DGAT1, mechanistic modeling, nonlinear mixed effect modeling, oral lipid tolerance test, pharmacodynamics, pharmacokinetics, triglyceride

## Abstract

We have developed a novel mechanistic pharmacokinetic‐pharmacodynamic (PK/PD) model to describe the time course of plasma triglyceride (TAG) after Oral Lipid Tolerance Test (OLTT) and the effects of AZD7687, an inhibitor of diacylglycerol acyltransferase 1 (DGAT1), in humans, rats, and mice. Pharmacokinetic and plasma TAG data were obtained both in animals and in two phase I OLTT studies. In the PK/PD model, the introduction of exogenous TAG is represented by a first order process. The endogenous production and removal of TAG from plasma are described with a turnover model. AZD7687 inhibits the contribution of exogenous TAG into circulation. One or two compartment models with first order absorption was used to describe the PK of AZD7687 for the different species. Nonlinear mixed effect modeling was used to fit the model to the data. The effects of AZD7687 on the plasma TAG time course during an OLTT as well as interindividual variability were well described by the model in all three species. Meal fat content or data from single vs repeated dosing did not affect model parameter estimates. Body mass index was found to be a significant covariate on the plasma TAG baseline. The system parameters of the model will facilitate analysis for other compounds and provide tools to bring the standard of OLTT data analysis closer to the analyses of Oral Glucose Tolerance Test data maximizing knowledge gain.

**Abbreviations**
AUC: area under the curve
AWERB: Animal Welfare Ethical Review Committee
DGAT1: diacylglycerol acyltransferase 1
FOCE‐ELS: first order conditional estimation
IPRED: individual predictions
MAD: Multiple Ascending Dose Study
OGTT: oral glucose tolerance test
OLTT: oral lipid tolerance test
OPTT: oral protein tolerance test
OPGLTT: oral protein‐glucose‐lipid tolerance test
PK: pharmacokinetic
PD: pharmacodynamic
QRPEM: expectation maximization method
SAD: Single Ascending Dose study
SMM: standardized mixed meal
TAG: triglyceride

## 1 INTRODUCTION

Metabolic challenge tests evaluate the body's ability to handle energy sources and are commonly used as health condition measures. The most commonly carried out tests are the Oral Glucose Tolerance Test (OGTT), the Oral Lipid Tolerance Test (OLTT), and the Oral Protein Tolerance Test (OPTT). Phenotypic flexibility has been postulated as a measure of health, suggesting that health can be evaluated by the ability to adapt to conditions of temporary stress and therefore the use of a standardized nutritional challenge test that combines properties of all three previously mentioned challenges into a combined oral protein‐glucose‐lipid tolerance test (OPGLTT) is expected to demonstrate subtle improvements of phenotypic flexibility, thereby enabling substantiation of nutritional health effects.^1^

In such a test, multi‐biomarker panels of interlinked measurements are generated, to fully evaluate and understand the results of such a test, a dynamic and quantitative understanding of the inter‐relation of the different components will be required. Before such a multi‐biomarker mathematical model can be developed, understanding of the dynamics of the key biomarkers of each of the three individual tests is required. The behavior of plasma glucose and insulin after an OGTT has been widely studied and nowadays can be successfully described mathematically.^2^, ^3^, ^4^, ^5^, ^6^ However, the same level of data analysis and modeling is lacking for plasma triglyceride (TAG) data after an OLTT.

Elevated levels of TAG are a major component of the metabolic syndrome and are important risk factors in the development of atherosclerotic cardiovascular disease.^7^ Measuring plasma TAG by means of an OLTT can determine the efficiency with which the individual uses lipid components.^8^ The analysis of the experimental data generated in an OLTT setting both in human and in animal studies vary substantially, from comparison of area under the curve (AUC) and/or the incremental area under the curve (iAUC),^9^ use of a 3‐point test,^10^ a scoring system,^7^ time‐matched comparison of single or multiple time‐points,^11^ comparison of the TAG clearance constants between groups^12^ and comparison of mean values during a defined time‐interval.^13^

These type of analyses have varied statistical validity to compare between different groups of one study^9^ and they also provide a pharmacodynamic measurement that sometimes has been used to establish PK/PD relationships when a xenobiotic have been used to modify the response compared to a control treated group.^14^ However, this analysis of the OLTT data does not provide sufficient granularity to allow for an equivalent level of knowledge gain from an OLTT study as the modeling of blood glucose and insulin data from an OGTT study delivers.^2^, ^3^, ^4^, ^5^, ^6^

A more mechanistic PK/PD modeling approach to mathematically describe the time course of the TAG excursion will provide the backbone for more meaningful analysis of OLTT data and in turn allowing for a deeper understanding of the potential action of different drugs on the postprandial lipid profile.

AZD7687 is a potent selective inhibitor of diacylglycerol acyltransferase 1 (DGAT1),^15^ which is involved in lipid absorption catalysing the final step in the biosynthesis of triacylglycerol. AZD7687 has been tested in several OLTT studies both in animals and in humans and multiple plasma TAG time courses are available Denison et al^14^, ^16^ and Barlind^15^ to date only very limited PK/PD analysis has been done on the data.

Data from the time course of the plasma TAG excursion and AZD7687 drug concentrations after an OLTT in humans, rats, and mice have been used to build a more mechanistic PK/PD model that describes the time course of plasma TAG following an OLTT. The results are summarized in this report.

## 2 MATERIALS AND METHODS

### 2.1 Data

#### 2.1.1 Human

Full details of the study protocols have previously been reported by Denison et al^14^ for Single Ascending Dose study (SAD) and Denison et al^16^ for the Multiple Ascending Dose Study (MAD). In brief, in the SAD, 80 healthy male subjects were enrolled. In each of 10 cohorts, six subjects received the same dose of AZD7687 orally (range across cohorts 1‐60 mg) and two placebo. Plasma AZD7687 exposure was measured repeatedly. Postprandial serum TAG excursion was measured during 8 hours after a standardized mixed meal (SMM) with fat energy content of either 60% or 45% before (baseline) and after dosing. Meal was served 4 hours after dosing of AZD7687. In the MAD, 62 overweight or obese healthy men were enrolled, of whom 42 received multiple doses of AZD7687 (1, 2.5, 5, 10, and 20 mg/day, n = 6 or n = 12 for each) and 20 received placebo for 1 week. Postprandial serum TAG was measured for 8 hours after a standardized 45% fat meal before compound dosing (baseline) and after the second and seventh daily dose of AZD7687. Meal was served 1 hour after dosing of AZD7687. Plasma AZD7687 exposure was measured repeatedly.

The studies were conducted in accordance with the ethical principles that have their origin in the Declaration of Helsinki and are consistent with the International Conference on Harmonization/Good Clinical Practice. Written informed consent was obtained from all subjects prior to initiation of any study procedures. Approval for conducting the SAD study was received by the Independent Ethics Committee, Mid Lands Institutional Review Board, KS, USA. Approval for conducting the MAD study was received by the independent National Research Ethics Service, St Thomas’ Hospital, London, UK.

#### 2.1.2 Rat

Full description of the OLTT in the rat has been published previously.^17^ In brief, 53 male Han‐Wistar rats (∼230 g), previously maintained on a chow diet, were fasted for 14 hours. Animals were dosed by oral gavage with either HPMC/Tween vehicle (n = 11) or AZD7687 suspended in HPMC/Tween at 0.1, 0.3, or 3 mg/kg (n = 12) per group and 2 hours later dosed with an oral gavage of corn oil (5 mL/kg). Six remaining animals were kept untreated and served as naive controls. N = 3 of the AZD7687 treated animals (per group) were serially bled for PK determination at 2, 3.5, 5, 8, and 11 hours post compound dose while the remaining animals on study where only bleed for compound concentrations 11 hours post compound dose to allow for serial blood collection for TAG measurement at 2, 2.5, 3.5, 5, 8, and 11 hours post compound dose.

#### 2.1.3 Mouse

Oral Lipid Tolerance Test in the mouse was carried out in a similar way to rats. In brief, 41 Male ICR mice (∼25 g) were dosed by oral gavage with either CMC vehicle (n = 8) or AZD7687 suspended in CMC at 0.1, 1, or 3 mg/kg (n = 11) per group and 0.5 hour later dosed with an oral gavage of fat emulsion containing 20% soybean oil (Intralipos 20%) at 10 mL/kg. N = 3 of the AZD7687‐treated animals (per group) were serially bled for PK determination at 1, 2, 3, and 4 hours post compound dose while the remaining animals on study where only bleed for TAG measurement at 0.5, 1.5, 2.5, 3.5, and 4.5 hours post compound dose.

All animal care and experimental procedures complied with the UK Home Office Animal Scientific Procedures Act 1986 and were approved by Animal Welfare Ethical Review Committee (AWERB). All studies involving animals are reported in accordance with the ARRIVE guidelines for reporting experiments involving animals.^18^, ^19^

### 2.2 The PK/PD model

**Figure 1:**
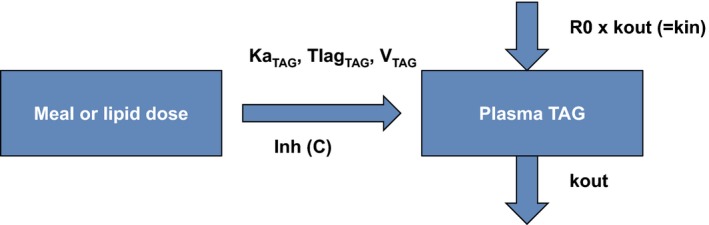
Schematic representation of the PK/PD model. Refer to equations 1‐4 for abbreviations

Biological plausibility combined with stable parameter estimation was used in the evaluation of different potential PK/PD models to help select the optimal one. The key components of the final PK/PD TAG model are described in Figure 1. The introduction of exogenous TAG into the system is represented by a first order process from the lipid depot compartment (assumed to be the gut) to the central compartment (plasma). A lag time to accommodate the time delay from ingestion of the fatty meal to the resulting increase in plasma TAG was needed. In addition, there is an endogenous production and removal of TAG from plasma described with a turnover model controlled by the turnover rate (kin) (production) and the fractional turnover rate (kout) (loss). Turnover model was reparameterized using the relationship of kin to baseline plasma TAG (R0) (R0 = kin/kout) to increase numerical stability.

Ka_TAG_ is the absorption rate of exogenous TAG into plasma, V_TAG_ is the volume of distribution of exogenous TAG, and Tlag_TAG_ is the time delay for the absorption of TAG from the SMM. The contribution of meal intake to the dynamics of TAG was incorporated into the model by considering the macronutrient composition of the meals. TAG from meals (clinical studies) in units of kcal was converted to grams of TAG input (9 kcal of fat is equivalent to 1 gr of TAG). The total energy content of the meal was 1100 kcal. The contribution of corn oil intake (rat studies) to the dynamics of TAG was incorporated into the model by assuming 100% of the corn oil was fat. Volume of oil administered was converted to grams applying a density value of 0.9 gr/mL. The contribution of soybean oil intake (mouse studies) to the dynamics of TAG was incorporated into the model by assuming 100% of the soybean oil was fat. Volume of soybean oil on the fat emulsion administered was calculated and converted to grams applying a density value of 0.917 gr/mL. It was assumed that all TAG administered was absorbed in the absence of AZD7687.

Based on the role of DGAT‐1, it is postulated that AZD7687 inhibits the contribution of exogenous TAG to circulation. A nonlinear relationship (equation (1)) provided a stable parameterization.

(1) $$\text{Inh (C)}=1-\frac{C}{C+{\text{IC}}_{50}},$$

where *C* is the AZD7687 concentration in plasma and IC_50_ is the concentration of compound that results in 50% of max inhibition.

The fixed structure of the final PK/PD model is shown in Figure 1 and its mathematical description on equations 2, 3, and 4. Residual error was estimated for plasma TAG using a proportional error model.

(2) $$\frac{{\text{dA}}_{\mathrm{TAG}}}{dt}=-{\text{ka}}_{\mathrm{TAG}}\times A_{\mathrm{TAG}},$$

(3) $${\text{EXOG}}_{\mathrm{TAG}}=\frac{A_{\mathrm{TAG}}}{V_{\mathrm{TAG}}},$$

(4) $$\frac{{\text{dC}}_{\mathrm{TAG}}}{dt}=R0\times \text{kout}+{\text{EXOG}}_{\mathrm{TAG}}\times {\text{ka}}_{\mathrm{TAG}}\times \text{Inh(C)}-\text{kout}\times C_{\mathrm{TAG}}.$$

C_TAG_ represents the plasma TAG concentration. Additionally, a lag time (Tlag_TAG_) to accommodate the time delay from ingestion of the fatty meal to the resulting increase in plasma TAG was applied to the A_TAG_ equation.

### 2.3 Pharmacokinetic models

Human PK was described with a 2‐compartment model with first order absorption, body weight was found to be a significant covariate for individual estimates of clearance (Cl) and volume of distribution (V). In the rat and mouse, as PK data were only collected for 4‐11 hours post dose, the plasma AZD7687 concentration data during the OLTT were characterized by 1‐compartment PK model. Full description of all the PK models is provided in supplementary section S1.

### 2.4 Modeling approach, implementation, and evaluation

A sequential approach of fitting the AZD7687 plasma concentrations first and then the plasma TAG secondly was used to select the structure of the models and get good initial estimates of the parameters. Subsequently, both AZD7687 plasma concentrations and plasma TAG levels were simultaneously fitted on the final analysis.

Models were implemented using nonlinear mixed effects modeling in Phoenix NLME, version 1.3 (Certara). The analysis was conducted using the first order conditional estimation (FOCE‐ELS) during the sequential and simultaneous analysis (the expectation maximization method (QRPEM) was used for the simultaneous fitting of the human data due to speed and ability to converge).

Interindividual variability was evaluated on all PK/PD parameters with an assumed log‐normal distribution of individual parameters. In addition, the potential effects on AZD7687 PK and plasma TAG of subject characteristics, such as age, body weight, height, age, BMI, and %fat on meal test were examined by scatterplots of individual parameter estimates vs covariates. Model selection was based on goodness of fit plots, the plausibility of the physiological system, and the objective function value provided by Phoenix NLME. A difference in the objective function value in hierarchical models of at least 10.83 (*P* = 0.001, assuming a chi‐squared distribution with 1 degree of freedom) was considered significant. Goodness of fit plots such as observed values vs population predictions (PRED) or vs individual predictions (IPRED), and conditional weighted residual errors vs time or vs predicted observations were used for graphic assessment of the quality of the model fit. The Phoenix NLME %CV reported was calculated using the Hessian method.

For the evaluation of the final models, 1000 data sets were simulated in Phoenix NLME. The median and the 95% prediction intervals of the individual concentration‐time profiles of AZD7687 and TAG were superimposed on the respective observed data.

### 2.5 AZD7687 plasma concentration

For all species, free plasma concentrations of AZD7687 were calculated based on measured concentrations of AZD7687 in plasma and corrected using a constant free fraction (fu) for each of the species. Free fraction values for AZD7687 across species have been previously published 15 (human fu = 0.055, rat fu = 0.03, mouse fu = 0.1). To be able to do cross species comparisons, modeling was carried out using free concentrations of AZD7687, therefore, in this report the term concentration of AZD767 refers to free concentration and resulting PK and PK/PD parameters obtained to describe the PK and TAG profiles were obtained using free concentrations of AZD7687 in plasma.

## 3 RESULTS

Plasma concentrations of AZD7687 in man were adequately described by a 2‐compartment model with first order absorption while in the rat and mice were described by 1‐compartment model with first order absorption. The pharmacokinetic parameters are shown in Table 1. The diagnostic plots and goodness of fit plots for the PK models are shown in supplementary section S1.

**Table 1:** Summary of the key PK and PK/PD parameters for AZD7687 in human, mouse, and rat (Full list of PK and PD parameters provided in Supplementary sections S1 and S2)

Parameter	Units	Human		Mouse		Rat	
		Value	% CV	Value	% CV	Value	% CV
Ka	1/hr	5.19	11.2	3	FIX	3	FIX
V	L	320.2	4.2	13.14	12.6	8.66	5.4
V2	L	136.8	10.2	–	–	–	–
Cl	L/hr	28.1	2.8	2.17	11.8	0.73	8.0
Cl2	L/hr	50.8	15.2	–	–	–	–
lag_tag	hr	1.63	2.2	0.98	0.5	1.24	5.6
Ka_tag	1/hr	0.74	15.3	2.10	15.8	0.37	28.5
V_tag	dL	250.4	14.5	0.012	28.6	2.19	25.2
R0	mg/dL	97.8	3.2	178.0	4.2	109.2	3.6
kout	1/hr	0.70	17.0	4.41	23.4	0.28	19.0
IC50	μmol/L	0.0078	35.3	0.081	33.1	0.162	74.3
IIV lag_tag	%	–	–	0.19	31.2	4.53	84.0
IIV Ka_tag	%	20.3	19.1	35.3	48.7	99.2	37.6
IIV V_tag	%	57.2	15.5	–	–	55.9	35.7
IIV R0	%	38.3	15.4	17.7	41.5	18.5	29.6
IIV kout	%	65.0	17.3	–	–	–	–
IIV IC50	%	223.4	15.4	62.0	71.3	–	–
TAG residual multiplicative		0.192	0.8	0.198	7.7	0.3	6.0

Effects of AZD7687 on the plasma TAG time course after SMM were well described by the PK/PD model proposed in all three species. The key estimated PK/PD parameters for all species are shown in Table 1.

### 3.1 Human data

The %fat of the different SMM tested did not significantly affect the model parameter estimates. No differences in the PK/PD relationship were observed between the single dose and multiple dose experiments. The resulting free in vivo IC_50_ was estimated to be 0.008 μmol/L. Interindividual variability in the IC_50_ was high (223%), additionally, interindividual variability was also estimated on ka_TAG_, V_TAG_, R0, and kout parameters according to a log‐normal distribution of individual parameters (Table 1). Inclusion of BMI as a covariate on the baseline plasma TAG levels (R0) significantly improved the model fit and made physiological sense (described in supplementary section S3). The lag time for the absorption of TAG from the meal test was estimated to be 1.6 hours which is in line with human stomach digestion times.

### 3.2 Rat data

The resulting free in vivo IC_50_ was 0.162 μmol/L**.** No interindividual variability could be estimated for IC_50_; however, it was possible for Tlag_TAG_, ka_TAG_, V_TAG_, and R0 parameters according to a log‐normal distribution of individual parameters (Table 1). The lag time for the absorption of TAG was 1.2 hours.

### 3.3 Mouse data

The resulting free in vivo IC_50_ (0.081 μmol/L) showed a 62% interindividual variability which is substantially lower than for human. Additionally, interindividual variability could also be estimated on Tlag_TAG_, ka_TAG_, and R0 parameters according to a log‐normal distribution of individual parameters (Table 1). The lag time for absorption of lipid was 0.98 hours.

**Figure 2:**
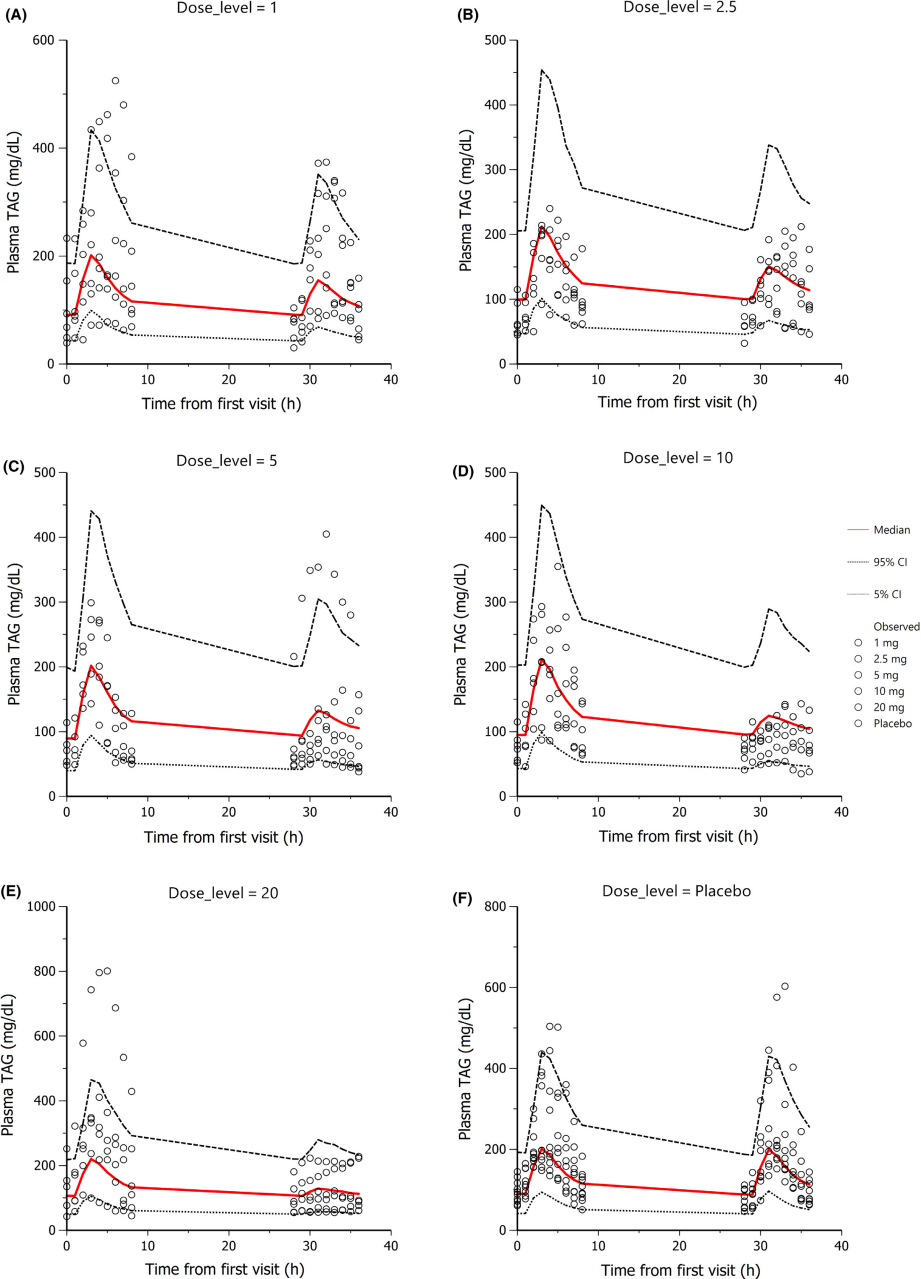
Goodness of fit plot for plasma TAG in human SAD study treated with 60% fat SMM. Plots available for all treatment groups in SAD and MAD in supplementary section S3. 1000 data sets were simulated. Red line is the median and dotted lines are the 95% prediction intervals of the individual plasma TAG‐time profiles. Observed data is plotted as dots. In the SAD study subjects were fed the 60% fat SMM at 0h (baseline) and 28h post the first visit. Plotted by dose level (1, 2.5, 5, 10, 20 mg and placebo)

**Figure 3:**
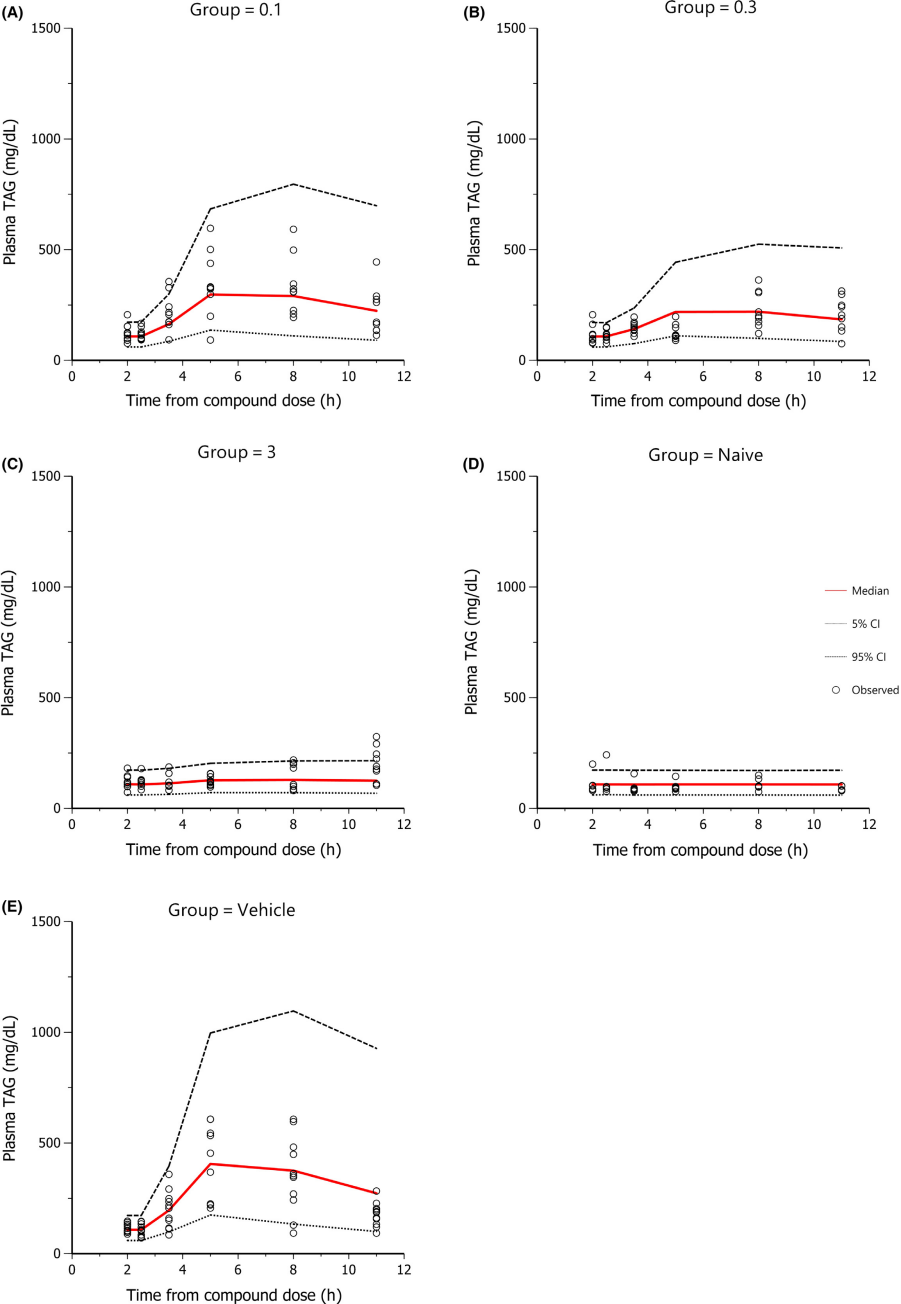
Goodness of fit plot for plasma TAG in rat. 1000 data sets were simulated. Red line is the median and dotted lines are the 95% prediction intervals of the individual plasma TAG‐time profiles. Observed data is plotted as dots. OLTT challenge (corn oil at 5 mL/kg) was performed 2h after compound dosing. Plotted by dose group (0.1, 0.3, 3 mg/kg, Naïve (i.e. no lipid challenge) and Vehicle)

**Figure 4:**
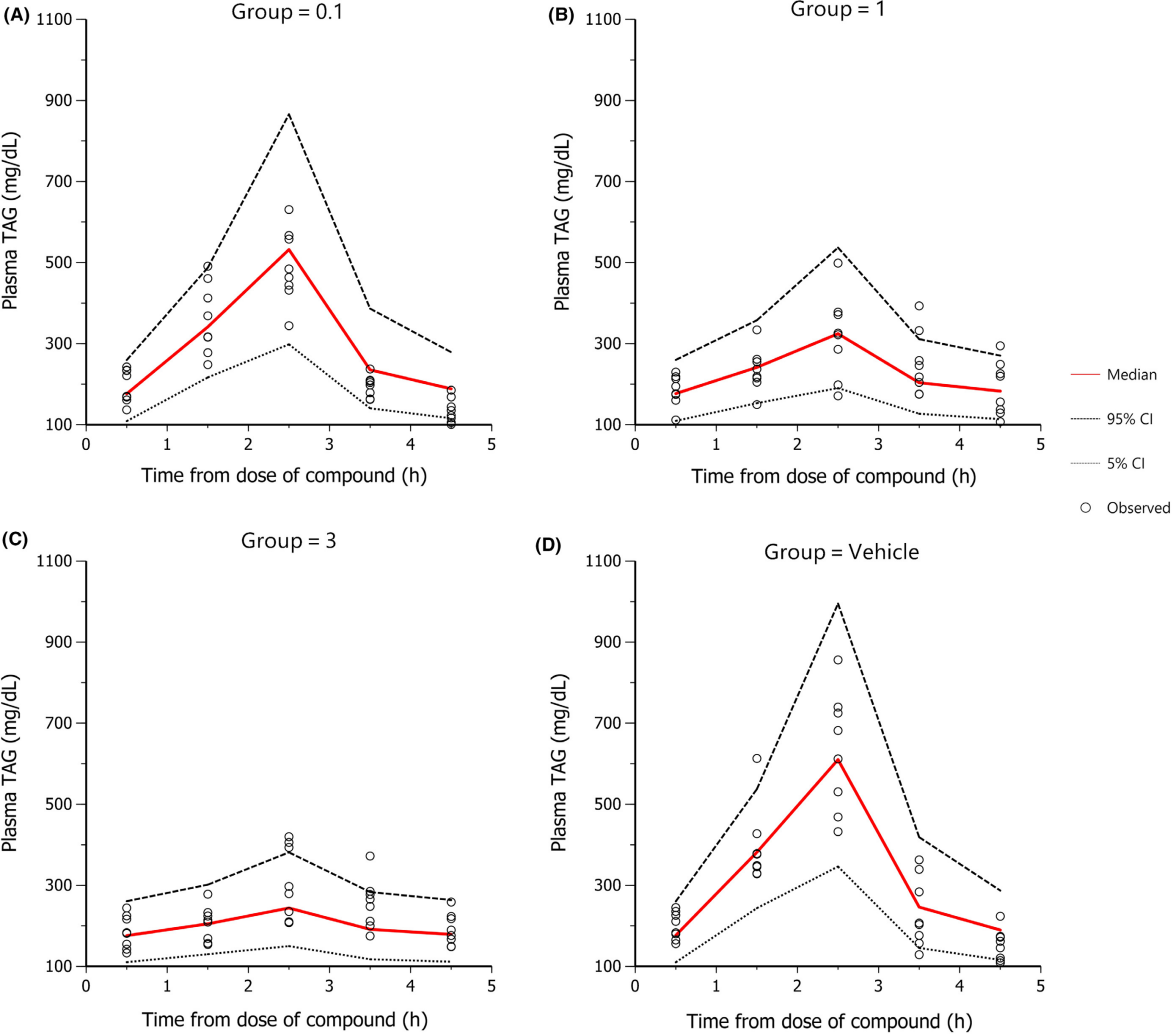
Goodness of fit plot for plasma TAG in mouse. 1000 data sets were simulated. Red line is the median and dotted lines are the 95% prediction intervals of the individual plasma TAG‐time profiles. Observed data is plotted as triangles. OLTT challenge (Intralipos 20% at 10 mL/kg) was performed 0.5h after compound dosing. Plotted by dose group (0.1, 1, 3 mg/kg, and Vehicle)

The median and the 95% prediction intervals of the individual plasma TAG profiles were superimposed on the respective observed data and are shown in Figures 2, 3, and 4 for human, rat, and mouse, respectively (for human, only a selection of doses are presented, all the doses fitted are shown in supplementary section S2). Diagnostic plots of the goodness of fit for all three species are shown in supplementary section S2.

## 4 DISCUSSION

An OLTT can determine the efficiency with which the individual handles lipid components such as TAG. The analysis of the experimental data generated in an OLTT setting vary substantially from comparison of area under the curve (AUC)^9^, ^20^, ^21^, ^22^, ^23^, ^24^, ^25^, ^26^, ^27^ and/or the incremental area under the curve (iAUC)^9^, ^10^, ^12^, ^22^, ^24^ use of a 3‐point test,^9^, ^10^ a scoring system,^8^ time‐matched comparison of single or multiple time‐points,^7^, ^11^, ^28^, ^29^, ^30^ comparison of the TAG clearance constants between groups^12^, ^31^ and comparison of mean values during a defined time‐interval.^13^

Despite OLTT been used as a surrogate test for potential new drugs in the metabolism area, very limited PK/PD modeling has been done to explore the effects that different concentrations of test compounds over time have in reducing the postprandial TAG excursion^16^, ^17^, ^29^, ^30^, ^32^, ^33^, ^34^, ^35^ The available PK/PD analysis of OLTT data found in the literature uses two types of TAG data depending on what has been collected during the OLTT (longitudinal data or single time point). However, both types of data sets are analyzed with similar direct response PK/PD models. Denison et al^14^ carried out PK/PD analysis of the plasma TAG iAUC measured during 8 hours post lipid load vs the plasma concentration of test compound at the start of the intake of the meal. Other authors^15^, ^36^, ^37^, ^38^ have carried out PK/PD analysis of a single time point plasma TAG level after a lipid challenge vs the plasma concentration of the drug at that same time point.

These types of limited PK/PD analysis do not maximize the potential learnings that can be extracted from the studies. Application of more mechanistic PK/PD modeling approaches to data generated on the analogue experiment OGTT has enhanced the level of understanding of the relationship between disease and response in such a challenge‐based study.^3^, ^5^, ^6^ Additionally, the application of more mechanistic modeling approaches has also provided important insights into the mechanism of action of drugs.^2^, ^4^ Similar kind of gains ought to be achieved as well from more mechanistic modeling of data generated in OLTT.

There are models published that describe drug‐induced changes in some plasma lipids in the absence of a lipid load^39^, ^40^, ^41^, ^42^, ^43^, ^44^ but to our knowledge none describe the plasma TAG excursion after an OLTT. In an OLTT setting, the “challenger” (fat load) can be considered the same as the pharmacodynamic response biomarker (plasma TAG) and therefore any successful mechanistic model will need to take into account the extra input of TAG simultaneously with the baseline data and the data during intervention of a TAG‐lowering compound. Such approach has previously successfully been used to describe plasma glucose concentrations over time after an OGTT.^2^, ^3^, ^4^, ^5^, ^6^ However, the above models had not been modified or applied to describe the plasma TAG time course following an OLTT challenge and therefore a gap still remained in the literature.

Clinical development of AZD7687,^15^ a novel DGAT1 inhibitor has recently been stopped due to intolerable side effects.^16^ DGAT1 is involved in the dietary absorption of TAG.^45^, ^46^, ^47^ Denison et al^14^, ^16^ have published the results from an OLTT carried out with AZD7687 in humans after single and multiple doses of AZD7687. However, the PK/PD analysis of that data was based on iAUC of the TAG excursion and the compound exposure at the time of fat loading. In addition, it has been previously published that AZD7687 reduces the TAG excursion after an OLTT in rats in an exposure dependant manner.^15^ Once again, the PK/PD analysis of that data was carried out using a single time point TAG level and the compound concentration at the same time point.

A novel semimechanistic PK/PD model has been proposed and evaluated in this report. The PK/PD model proposed describes well the magnitude and time course of change on the plasma TAG excursion during an OLTT after treatment with AZD7687 across three species. It provides a numerical quantification of the “in vivo” potency of the drug allowing robust comparison of the efficacy of AZD7687 across species. Estimated in vivo IC_50s_ are in good agreement with the previously published values in human (0.02 μmol/L from the AZD7687 SAD OLTT study using iAUC analysis of the plasma TAG vs plasma concentration at the start of the meal test^14^ and in rat (0.042 μmol/L for rat AZD7686 OLTT study using single time point data for plasma TAG and PK^15^). Human interindividual variability on IC_50_ was high (223%), but this is a general observation also noticed for other drugs in the cardiovascular and metabolic area in the literature^42^, ^48^, ^49^, ^50^, ^51^ and reflects the heterogeneity of the human population. Interindividual variability on IC_50_ was lower for mouse. Similarly, interindividual variability in TAG baseline levels (R0) was higher in human (38%) compared to mouse (18%) and rat (18%) reflecting the expected larger heterogeneity between humans and lab animals.

In vitro potency (IC_50_) for AZD7687 against DGAT1 (in liver microsomes) has been published previously (Barlind_2012) in human (0.07 μmol/L), rat (0.15 μmol/L), and mouse (0.1 μmol/L). While there is very good agreement between in vitro and in vivo values across rat and mouse, AZD7687 has shown to be more potent in vivo in the OLTT (compared to the in vitro value) in humans. AZD7687 has shown to be more potent in vitro (0.01 μmol/L) in HuTu 80 (Human gastrointestinal tumor cell line) compared to human liver microsomes (Barlind_2012); the estimated in vivo potency in humans is also in very good agreement with the in vitro HuTu 80 value suggesting that the differences between in vitro and in vivo in man could be explained for differences in affinity toward DGAT1 in different organs. Unfortunately, there is no rodent equivalent to HuTu 80 to test AZD7687 in vitro to validate this hypothesis. It is however worth mentioning that circulating free plasma concentrations of AZD7687 have been used as surrogate for the modeling while the major target organ during OLTT is DGAT1 in the intestine, and thus AZD7687 exposure in the gut could potentially have been higher than in circulation at the time of the fat challenge and therefore more aligned with the microsomal IC_50_. However, a comparison of the obtained individual IC_50_ for human subjects in the SAD and MAD study showed no difference between both studies even though the SMM was given 4 hours post dose in the SAD study and 1 hour post dose in the MAD study. It is expected that for a given plasma concentration, the concentrations in the enterocyte at those time points will be different and therefore lead to different IC_50_. It is worth mentioning that AZD7687 is rapidly absorbed with *T*_max_ achieved in plasma within 1 hour post dose and therefore the comparison between those two time points (ie, 1 and 4 hours post dose) might not be the most relevant one to do to test the hypothesis. Further work in the future using a more mechanistic PK model of the gut may provide additional information on this respect.

One complexity when tackling mathematical modeling of OLTT is that contrary to OGTT, where the challenge agent is the same analyte that is monitored in plasma during the excursion, in an OLTT, the challenge agents are lipids that needs to get digested and resynthesized into TAG. Depending on the degree of saturation of the lipids, their preference for packing into TAG or other species like cholesterol varies.^52^ Furthermore, the length of the lipids acyl chains, the position of the double bonds, and the conformation of the double bonds determine the metabolic fate of dietary fat digestion and absorption^53^, ^54^, ^55^ resulting on potentially different postprandial TAG excursions for same amount of fat of different lipid composition.^56^, ^57^ In this study, different lipid challenge agents are used (a mixed meal in humans, corn, or soybean oil in preclinical species) expecting different resulting postprandial TAGs. The PK/PD model developed describes the dynamics of the TAG excursion only considering the macronutrient composition of the meal and assuming all fat content is converted into TAG. Therefore, the resulting TAG absorption‐related parameters of the model (Ka_TAG_, Tlag_TAG_, and V_TAG_) not only represent physiological differences across species but also are specific for the type of fat challenge administered to each species and therefore their use on prospective experiments under different challenge conditions will need to be carefully considered. Nevertheless they should be adequate starting values for rapid parameter estimation for other OLTT experiments with different lipid challenge compositions if needed.

In the PK/PD model, it is assumed that the DGAT1 inhibitor can only affect the absorption of the lipid administered on the meal test and not any TAG already prepacked in the enterocyte from earlier meals. In addition, the model assumes that in the absence of the DGAT1 inhibitor, 100% of the administered fat is absorbed which is consistent with the reduced TAG levels present in feces^58^ however, the lack of TAG in the feces does not mean that all the TAG dosed reached the plasma within 8 h post dose, as evidenced by the very early TAG peaks (~20 minutes post meal) sometimes observed in an OLTT^59^ and presumed to be from a previous meal. A very extensive blood sampling is required to fully quantify this first peak and one needs to carefully consider if this additional cost and complication on a clinical OLTT study will be worth based on the knowledge of the mechanisms the potential drugs are modulating and the overall impact on the OLTT excursion of any plasma TAG peaks coming from a previous meal. With our sampling protocol, we were able to fully detect a different TAG excursion on subjects receiving a 60% fat content mixed meal and those receiving 45% indicating that it was sufficient to describe the postprandial TAG excursion and therefore study the effect of DGAT1i. The PK/PD model however includes an additional pathway for TAG to appear in plasma independent of the meal challenge that could encompass multiple routes such as release of existing TAG in the enterocyte and other tissues, so it could be modified if any of those areas are of relevance to other targets. For our work, it is postulated that in the short term of the OLTT, DGAT1i does not have any impact on those other processes.

Ables et al^7^ have nicely demonstrated that in animal models while DGAT1i‐induced gastric retention can play a role in the acute inhibition of chylomicron secretion, it is not necessary for the effects of DGAT1i on postprandial TAG excursions. In addition, Denison et al^16^ demonstrated very limited and transient effects of AZD7687 on paracetamol absorption in man as a marker of delayed gastric emptying, with paracetamol AUC in plasma captured completely within 8 hours post dose and concluded that it was unlikely that delayed gastric emptying contributed substantially to the decrease in TAG excursion. Therefore, a rise of plasma TAG at later time points (>8 hours) was very unlikely and the monitoring of plasma TAG for the selected time points post dose is believed to be sufficient to study the effects of DGAT1 inhibition on postprandial TAG. Obviously, the same might not be true for other mechanisms other than DGAT1i. In such cases, the PK/PD model presented here can be easily modified to incorporate different mechanisms of action such as delaying or slowing lipid absorption through compound driven effects on Tlag_TAG_ and Ka_TAG_, respectively.

Taking into consideration the above‐mentioned potential model modifications needed to accommodate different study designs and modes of actions, this model will enable similar kind of analysis for other compounds. Additional mechanistic modifications to the model should also be evaluated to explain the augmented TAG response to an OLTT in several conditions such as obesity, T2DM, and nonalcoholic steatohepatitis.^1^ Expansion of the model to include additional biomarkers describing other lipid metabolism effects such as lipolysis, adipokine production, and lipoprotein production that are also triggered by an OLTT should be attempted.

In conclusion, this model provides not only a description of the effects of AZD7687 on plasma TAG in an OLTT setting across all three species but also the tools to start bringing the standard of OLTT data analysis closer to how OGTT data are analyzed maximizing the knowledge gain and the possibility to compare results between the tests. In the future, this model could contribute to the difficult task of building a cross‐biomarker model that can help explain the multi‐variable results obtained in OPGLTT.

## AUTHOR CONTRIBUTIONS

PMG, JY, and CN were involved in development of study concept, analysis, and interpretation of the data. SB and PMG designed the animal studies and SB collected the animal data. All authors contributed to the drafting and review of the manuscript.

## DISCLOSURES

All authors were employees at AstraZeneca at the time the studies were carried out.
