# Supplementary material for: Evolving data analysis of an Oral Lipid Tolerance Test toward the standard for the Oral Glucose Tolerance Test: Cross species modeling effects of AZD7687 on plasma triacylglycerol
Source: Pharmacol Res Perspect. 2019 Mar 9;7(2):e00465. doi: 10.1002/prp2.465 (PMC6408865; doi:10.1002/prp2.465)
Supplement: Supplementary file 1 [file PRP2-7-e00465-s001.docx]

**Supplementary section S1**

**PK fitting in human**

A 1 compartment PK model was initially tested but the plasma AZD7687 concentration data were better characterized by a two-compartment PK model parameterized in terms of absorption rate constant (ka), apparent clearance (CL), the central volume of distribution (V1), distributional clearance (CL_D_) and the peripheral volume of distribution (V2). Inter-individual variability was estimated on CL, V1, V2 and ka parameters according to a log-normal distribution of individual parameters. Body Weight (BW) was found to be a significant covariate for individual estimates of CL, V1 and V2. The final equations for CL, V1 and V2 are [eq 1], [eq 2] and [eq 3] respectively. Correlation between Cl and V1 parameters was allowed as it reduced the objective function significantly. Residual error was characterized with a proportional plus additive error model.

${Cl}_{i}=tvCL\times{(\frac{BW}{mean \left( BW \right)})}^{dCldWeight}\times\exp({nCl}_{i})$ [eq 1]

${V1}_{i}=tvV1\times{(\frac{BW}{mean \left( BW \right)})}^{dV1dWeight}\times\exp({nV1}_{i})$ [eq 2]

${V2}_{i}=tvV2\times{(\frac{BW}{mean \left( BW \right)})}^{dV2dWeight}\times\exp({nV2}_{i})$ [eq 3]

Where BW is centred at its mean (85.3 kg), tvCl, tvV1 and tvV2 are the population values, dCldWeight, dV1dWeight and dV2dWeight are the exponent on body weight and nCl, nV1 and nV2 are the inter-individual variability parameters for Cl, V1 and V2 respectively.

The resulting pharmacokinetic parameters are shown in Table 1. Diagnostic plots of the goodness of the fit are shown in Fig 1. 1000 data sets were simulated in Phoenix NLME. The median and the 95% prediction intervals of the individual concentration–time profiles of AZD7687 were superimposed on the respective observed data (Fig 2).

**Table 1:** Human PK parameters for AZD7687 (using free concentrations in plasma). IIV (Inter-Individual variability)

| Parameter | Value | Units | %CV |
| --- | --- | --- | --- |
| tvka | 5.19 | 1/hr | 11.2 |
| tvV1 | 320.2 | L | 4.2 |
| tvV2 | 136.8 | L | 10.2 |
| tvCL | 28.1 | L/hr | 2.8 |
| tvCLD | 50.8 | L/hr | 15.2 |
| dCLdWeight | 1.05 |  | 14.3 |
| dV1dWeight | 1.18 |  | 23.9 |
| dV2dWeight | 1.89 |  | 36.1 |
| IIV CL | 21.8 | % | 16.2 |
| IIV V1 | 19.4 | % | 15.1 |
| IIV ka | 62.4 | % | 18.2 |
| IIV V2 | 47.0 | % | 18.0 |
| Cl-V1 Correlation | 0.59 |  |  |
| Conc Residual Additive | 0.0005 | uM | 1.7 |
| Conc Residual Multiplicative | 0.159 |  | 1.0 |

**Figure 1:** Goodness of fit plots for plasma concentrations of AZD7687 in man. A) Observed data (DV) versus individual predicted (IPRED) values. B) Observed data (DV) versus population predicted (PRED) values C) Conditional Weighted Residuals (CWRES) versus time (IVAR). D) Conditional Weighted Residuals (CWRES) versus predicted values (PRED)

| A  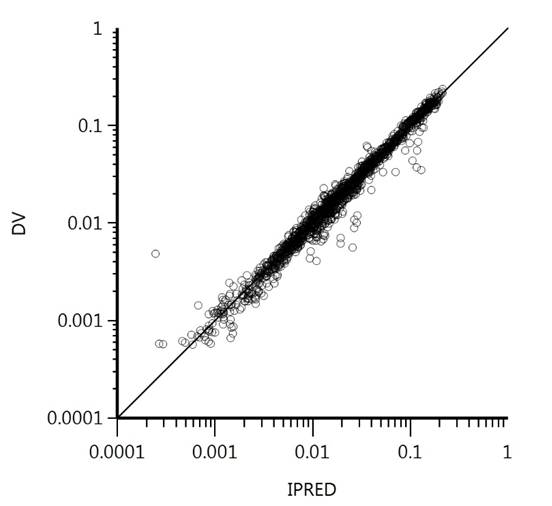 | B  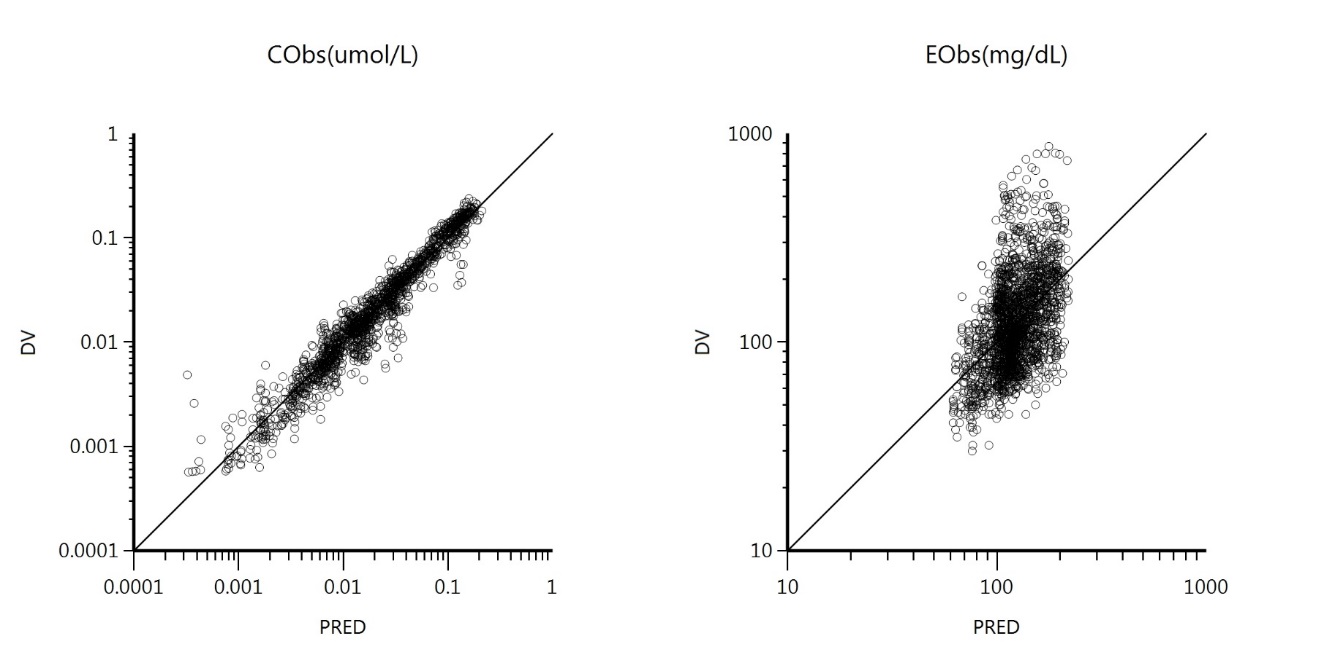 |
| --- | --- |
| C  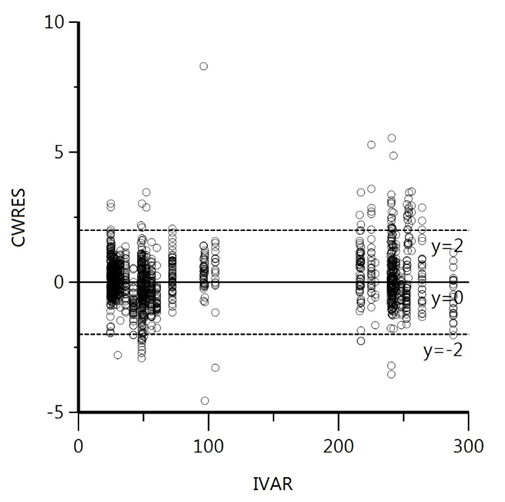 | D  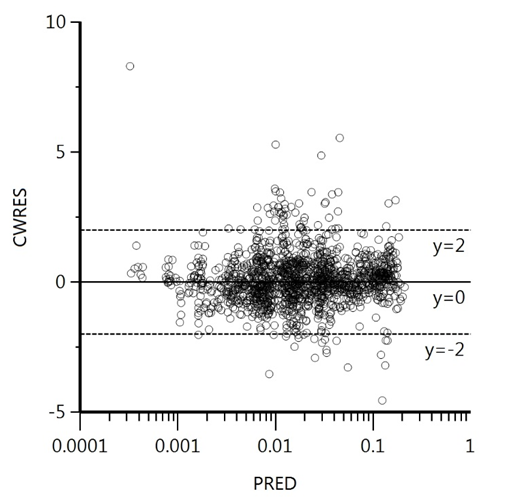 |

**Figure 2:** Goodness of fit plot for plasma concentrations for AZD7687 in human. 1000 data sets were simulated. Blue line is the median and dotted lines are the 95% prediction intervals of the individual concentration–time profiles of AZD7687. Observed data is plotted as dots. Graphs are separated by study (SAD and MAD), fat content of the SMM (60% and 45%) as well as dose level. SAD study was a single dose of AZD7687 24h post first visit and MAD study had doses at 48, 96, 120, 144, 168, 192, 216 and 240h post first visit (apart from 2.5 mg bid dose group that had doses at 48, 96, 108, 120, 132, 144, 156, 168, 180, 192, 204, 216, 228, 240 and 252h). A, B) SAD with 45% fat SMM at 5 and 20 mg respectively; C-G) SAD with 60% fat SMM at 1, 2.5, 5, 10 and 20 mg respectively; H-L) MAD with 45% fat SMM at 1, 2.5, 2.5 mg bid, 5 and 20 mg respectively.

| A   | B   | C  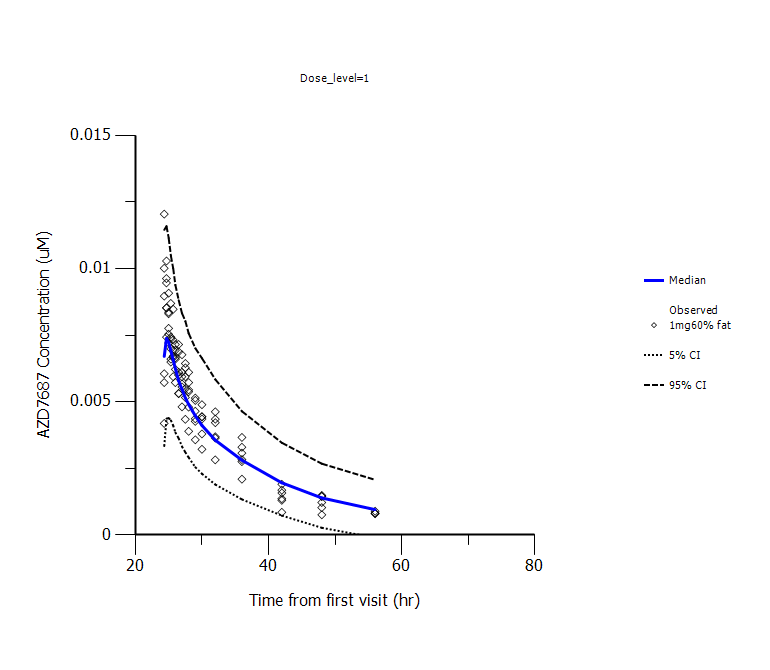 |
| --- | --- | --- |
| D   | E   | F   |
| G   | H   | I   |
| J   | K   | L   |

**PK fitting in rat**

As PK data was only collected for 11h post dose, the ability to fit complex PK models was limited. The aim of the PK fitting was to describe the kinetics of the compound during the OLTT instead of a full characterization of the PK properties of the compound. The plasma AZD7687 concentration data during the OLTT were characterized by one-compartment PK model parameterized in terms of absorption rate constant (ka), apparent clearance (CL) and apparent volume of distribution (V). Inter-individual variability was estimated on CL and V parameters according to a log-normal distribution of individual parameters. A fixed absorption rate of 3h^-1^ had to be assumed because there was no PK data collected the first 2h post compound dose and therefore ka could not be estimated. However, resulting CL and V parameters were in good agreement with previously published values [16] when corrected for protein binding. Additionally, sensitivity analysis of a range of values of ka showed it had no impact on other parameter estimates. Residual error was characterized with a proportional plus additive error model.

Plasma concentrations of AZD7687 were adequately described by a 1-compartment model and the Pharmacokinetic parameters are shown in Table 2. Diagnostic plots of the goodness of the fit are shown in Fig 3. 1000 data sets were simulated in Phoenix NLME. The median and the 95% prediction intervals of the individual concentration–time profiles of AZD7687 were superimposed on the respective observed data (Fig 4).

**Table 2:** Rat PK parameters for AZD7687 (using free concentrations in plasma). IIV is Inter-Individual variability.

| Parameter | Value | Units | %CV |
| --- | --- | --- | --- |
| tvKa | 3 | 1/hr | FIXED |
| tvV | 8.66 | L/kg | 5.4 |
| tvCl | 0.73 | L/(kg*hr) | 8.0 |
| IIV V | 11.7 | % | 81.3 |
| IIV CL | 45.4 | % | 30.1 |
| Conc Residual Additive | 0.0023 | uM | 41.0 |
| Conc Residual Multiplicative | 0.15 |  | 15.2 |

**Figure 3:** Goodness of fit plots for plasma concentrations of AZD7687 in the rat. A) Observed data (DV) versus individual predicted (IPRED) values. B) Observed data (DV) versus population predicted (PRED) values C) Conditional Weighted Residuals (CWRES) versus time (IVAR). D) Conditional Weighted Residuals (CWRES) versus predicted values (PRED)

| A  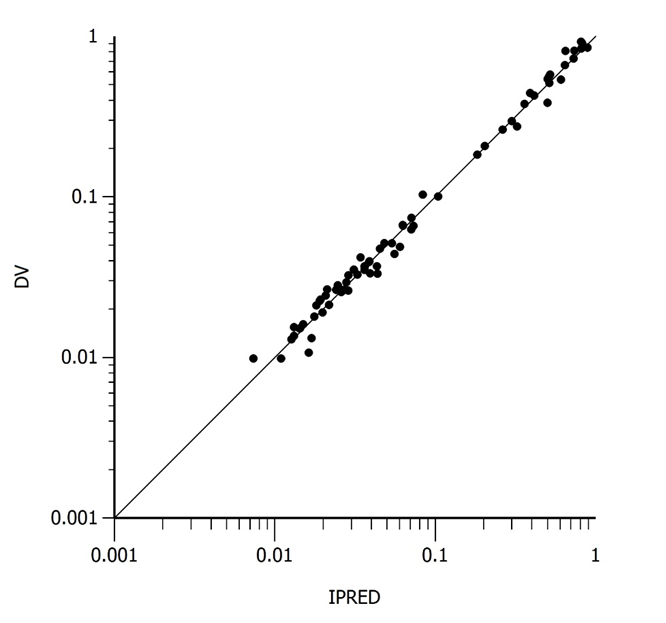 | B  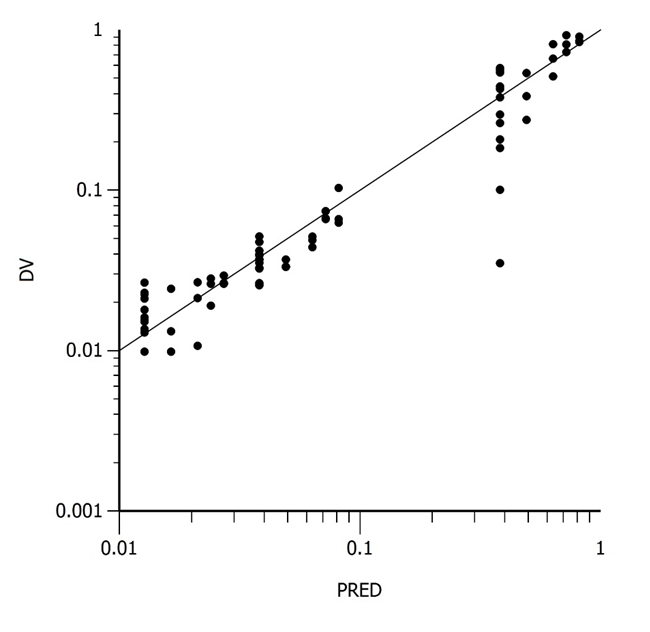 |
| --- | --- |
| C  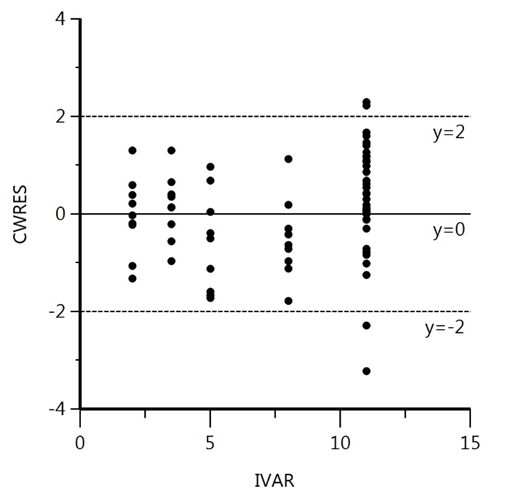 | D  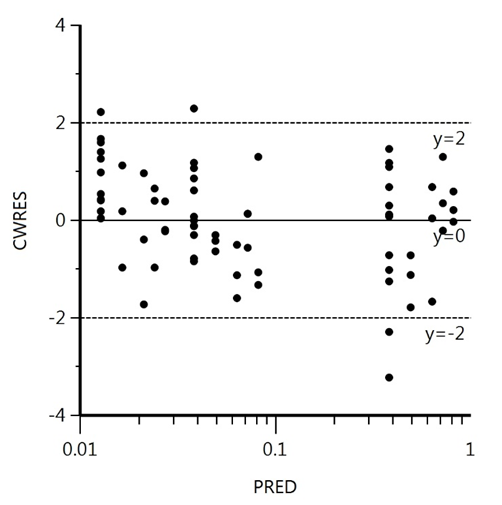 |

**Figure 4:** Goodness of fit plot for plasma concentrations for AZD7687 in rat. 1000 data sets were simulated. Blue line is the median and dotted lines are the 95% prediction intervals of the individual concentration–time profiles of AZD7687. Observed data is plotted as triangles. A) 0.1 mg/kg dose; B) 0.3 mg/kg dose; C) 3 mg/kg dose.

| A   | B   | C   |
| --- | --- | --- |

**PK model in mouse**

As PK data was only collected for 4h post dose, the ability to fit complex PK models was limited. As with the rat, the aim of the PK fitting was to describe the kinetics of the compound during the OLTT instead of a full characterization of the PK properties of the compound. The plasma AZD7687 concentration data during OLTT were characterized by one-compartment PK model parameterized in terms of absorption rate constant (ka), apparent clearance (CL) and apparent volume of distribution (V). Inter-individual variability was estimated on CL and V parameters according to a log-normal distribution of individual parameters. Correlation between Cl and V parameters was allowed. A fixed absorption rate of 3h^-1^ had to be assumed because there was no PK data collected the first 1h post compound dose and therefore could not be estimated. However, resulting CL and V parameters were in good agreement with previously published values [16] when corrected for protein binding. Additionally, sensitivity analysis of a range of values of ka showed it had no impact on other parameter estimates. Residual error was characterized with a proportional error model.

Plasma concentrations of AZD7687 were adequately described by a 1-compartment model and the Pharmacokinetic parameters are shown in Table 3. Diagnostic plots of the goodness of the fit are shown in Fig 5. 1000 data sets were simulated in Phoenix NLME. The median and the 95% prediction intervals of the individual concentration–time profiles of AZD7687 were superimposed on the respective observed data (Fig 6).

**Table 3:** Mouse PK parameters for AZD7687 (using free concentrations in plasma). IIV is Inter-Individual variability.

| Parameter | Value | Units | %CV |
| --- | --- | --- | --- |
| tvKa | 3 | 1/hr | FIXED |
| tvV | 13.14 | L/kg | 12.6 |
| tvCl | 2.17 | L/(kg*hr) | 11.8 |
| IIV V | 27.7 | % | 37.8 |
| IIV CL | 35.9 | % | 37.8 |
| Cl-V Correlation | 0.419 |  |  |
| Conc Residual Multiplicative | 0.112 |  | 15.8 |

**Figure 5:** Goodness of fit plots for plasma concentrations of AZD7687 in the mouse. A) Observed data (DV) versus individual predicted (IPRED) values. B) Observed data (DV) versus population predicted (PRED) values C) Conditional Weighted Residuals (CWRES) versus time (IVAR). D) Conditional Weighted Residuals (CWRES) versus predicted values (PRED)

| A  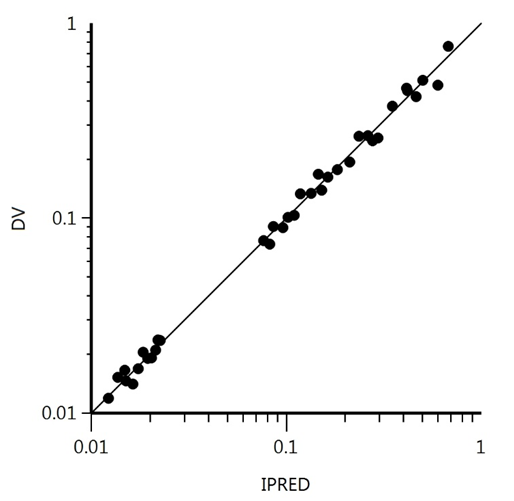 | B  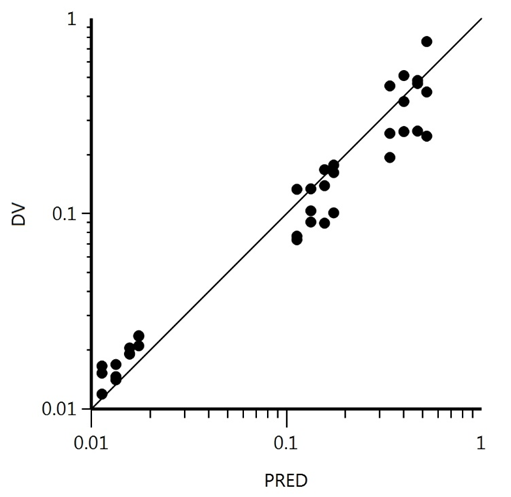 |
| --- | --- |
| C  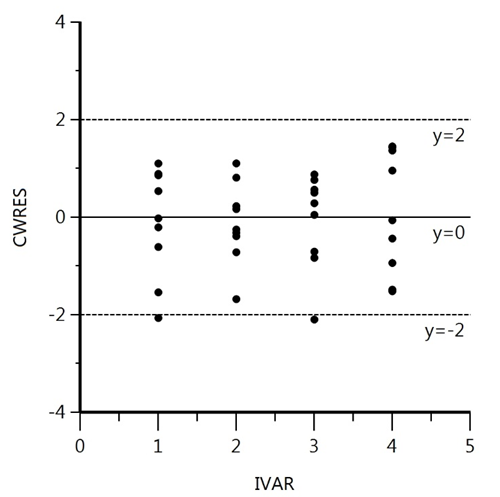 | D  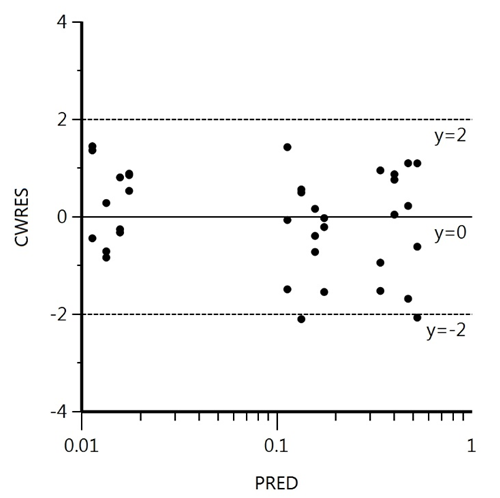 |

**Figure 6:** Goodness of fit plot for plasma concentrations for AZD7687 in mouse. 1000 data sets were simulated. Blue line is the median and dotted lines are the 95% prediction intervals of the individual concentration–time profiles of AZD7687. Observed data is plotted as dots. A) 0.1 mg/kg dose; B) 1 mg/kg dose; C) 3 mg/kg dose.

| A   | B   | C   |
| --- | --- | --- |

**Supplementary section S2**

**Human PK/PD modelling**

Inter-individual variability was estimated on ka_TAG_, V_TAG_, R0, IC_50_ and kout parameters according to a log-normal distribution of individual parameters. BMI was found to be a significant covariate for individual estimates of plasma TAG baseline (R0) [eq 4].

$R0=tvR0\times{(\frac{BMI}{mean \left( BMI \right)})}^{dR0dBMI}\times\exp({nR0}_{i})$ [eq 4]

Where BMI is centred at its mean (27.5 kg/m^2^), tvR0 is the population value, dR0dBMI is the exponent on BMI and nR0 is the inter-individual variability parameter for R0

Plasma TAG concentrations were adequately described by the PK/PD model and the PK/PD parameters are shown in Table 4. Diagnostic plots of the goodness of the fit are shown in Fig 7. 1000 data sets were simulated in Phoenix NLME. The median and the 95% prediction intervals of the individual TAG concentration–time profiles were superimposed on the respective observed data (Fig 8).

**Table 4:** Human PK/PD parameter Estimates for effects of AZD7687 in Plasma TAG following OLTT. IIV is Inter-Individual variability.

| Parameter | Value | Units | %CV |
| --- | --- | --- | --- |
| tvTlag_TAG_ | 1.63 | hr | 2.2 |
| tvKa_TAG_ | 0.74 | 1/hr | 15.3 |
| tvV_TAG_ | 250.4 | dL | 14.5 |
| tvR0 | 97.8 | mg/dL | 3.2 |
| tvkout | 0.70 | 1/hr | 17.0 |
| tvIC_50_ | 0.0078 | umol/L | 35.3 |
| dR0dBMI | 1.28 |  | 17.0 |
| IIV Ka_TAG_ | 20.3 | % | 19.1 |
| IIV V_TAG_ | 57.2 | % | 15.5 |
| IIV R0 | 38.3 | % | 15.4 |
| II kout | 65.0 | % | 17.3 |
| IIV IC_50_ | 223.4 | % | 15.4 |
| TAG Residual Multiplicative | 0.192 |  | 0.8 |

**Figure 7:** Goodness of fit plots for plasma TAG in human. A) Observed data (DV) versus individual predicted (IPRED) values. B) Observed data (DV) versus population predicted (PRED) values C) Conditional Weighted Residuals (CWRES) versus time (IVAR). D) Conditional Weighted Residuals (CWRES) versus predicted values (PRED)

| A  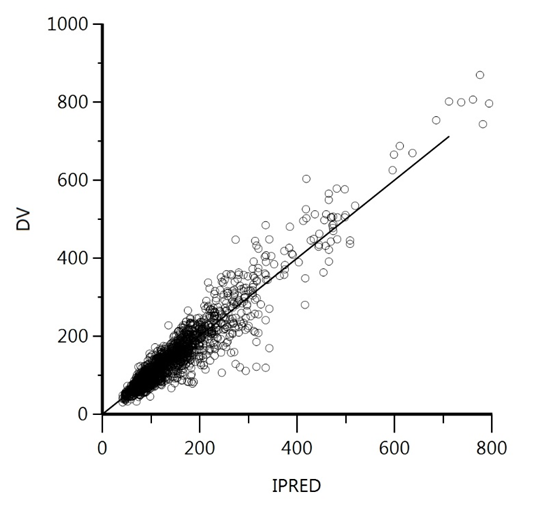 | B  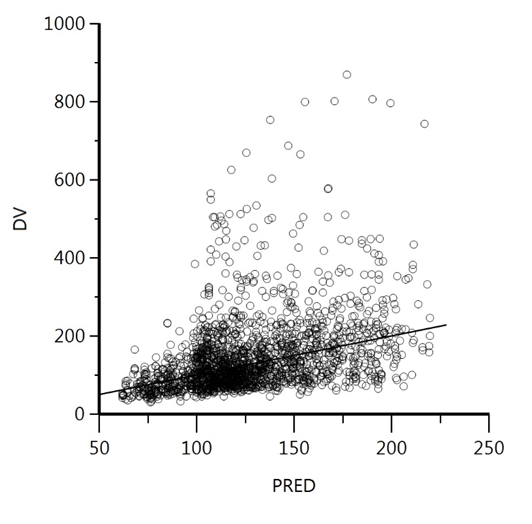 |
| --- | --- |
| C  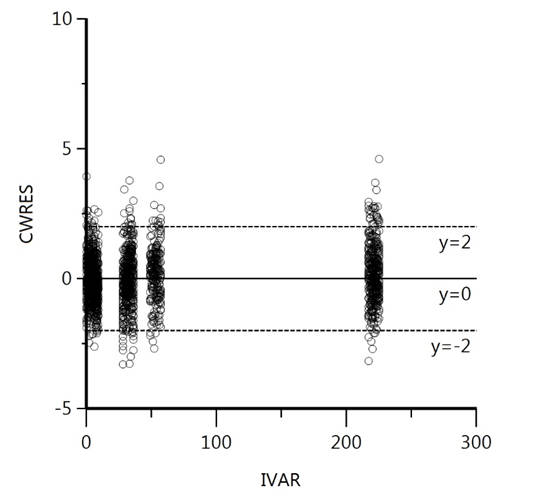 | D  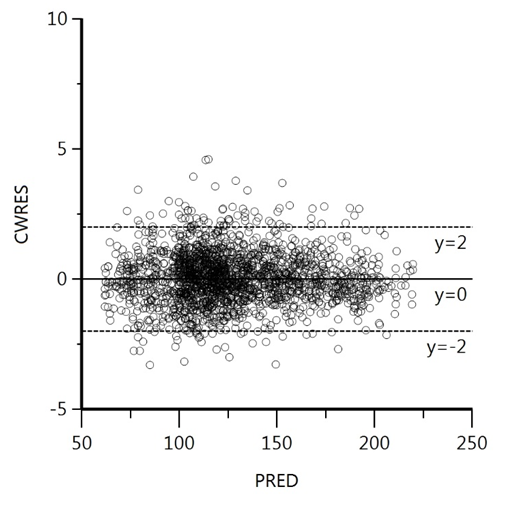 |

**Figure 8:** Goodness of fit plot for plasma TAG in human. 1000 data sets were simulated. Red line is the median and dotted lines are the 95% prediction intervals of the individual plasma TAG–time profiles. Observed data is plotted as dots. Graphs are separated by study (SAD and MAD), fat content of the SMM (60% and 45%) as well as dose level. In addition, MAD groups have their SMM challenge days (under drug treatment) in separated plots for ease of visualization. In the SAD study subjects were fed the SMM at 0h (baseline) and 28h post the first visit. In the MAD study subjects were fed the 45% SMM at 0h (baseline), 49h and 217h after the first visit (2.5mg groups not challenge at 49h). A-C) SAD with 45% fat SMM at 0, 5, and 20 mg respectively; D-I) SAD with 60% fat SMM at 0, 1, 2.5, 5, 10 and 20 mg respectively; J1-J3) MAD (Placebo) with 45% fat SMM; K1-K3) MAD (1 mg) with 45% fat SMM; L1-L2) MAD (2.5 mg) with 45% fat SMM; M1-M2) MAD (2.5 mg bid) with 45% fat SMM; N1-N3) MAD (5 mg) with 45% fat SMM; O1-O3) MAD (20 mg) with 45% fat SMM.

| A   | B   | C   |
| --- | --- | --- |
| D   | E   | F   |
| G   | H   | I   |
| J1   | J2 (time Zoom of J1)   | J3 (time Zoom of J1)   |
| K1   | K2 (time Zoom of K1)   | K3 (time Zoom of K1)   |
| L1   |  | L2 (time Zoom of L1)   |
| M1   |  | M2 (time Zoom of M1)   |
| N1   | N2 (time Zoom of N1)   | N3 (time Zoom of N1)   |
| O1   | O2 (time Zoom of O1)   | O3 (time Zoom of O1)   |

**Rat PK/PD modelling**

The same structural model that was used for the analysis of the human plasma TAG data was used for the analysis of the rat data. Inter-individual variability was included on ka_TAG_, Tlag_TAG_, V_TAG_ and R0 parameters according to a log-normal distribution of individual parameters. Inclusion/Exclusion of inter-individual variability in the different parameters was guided by quality of the model output and evaluation of the objective function. Residual error was estimated for plasma TAG using a proportional error model.

Effects of AZD7687 in the plasma TAG time course after SMM was well described by the PK/PD model proposed. The estimated PK/PD parameters are shown in Table 5. Diagnostic plots of the goodness of the fit are shown in Fig 9.

**Table 5:** Rat PK/PD parameter Estimates for effects of AZD7687 in Plasma TAG following OLTT. IIV is Inter-Individual variability.

| Parameter | Value | Units | %CV |
| --- | --- | --- | --- |
| tvTlag_TAG_ | 1.24 | hr | 5.6 |
| tvKa_TAG_ | 0.37 | 1/hr | 28.5 |
| tvV_TAG_ | 2.19 | dL | 25.2 |
| tvR0 | 109.3 | mg/dL | 3.6 |
| tvkout | 0.28 | 1/hr | 19.0 |
| tvIC_50_ | 0.162 | umol/L | 74.3 |
| IIV lag_TAG_ | 4.53 | % | 84.0 |
| IIV Ka_TAG_ | 99.2 | % | 37.6 |
| IIV V_TAG_ | 55.9 | % | 35.7 |
| IIV R0 | 18.5 | % | 29.6 |
| TAG Residual Multiplicative | 0.3 |  | 6.0 |

**Figure 9:** Goodness of fit plots for plasma TAG in rat. A) Observed data (DV) versus individual predicted (IPRED) values. B) Observed data (DV) versus population predicted (PRED) values C) Conditional Weighted Residuals (CWRES) versus time (IVAR). D) Conditional Weighted Residuals (CWRES) versus predicted values (PRED)

| A  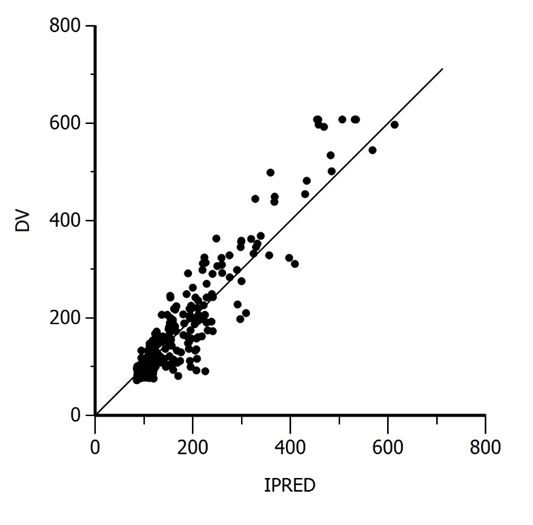 | B  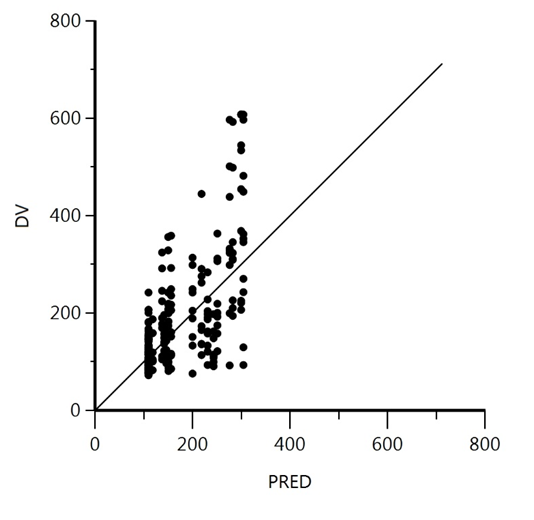 |
| --- | --- |
| C  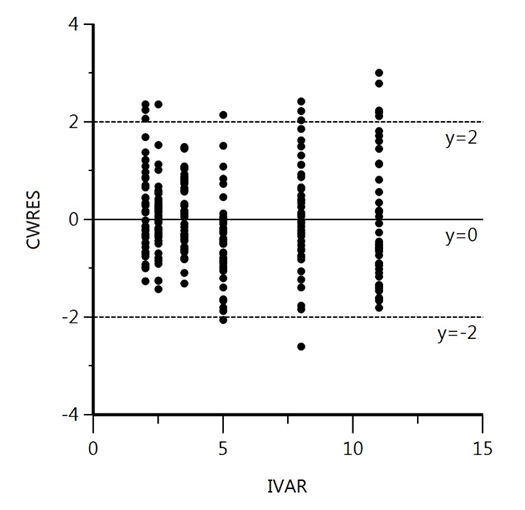 | D  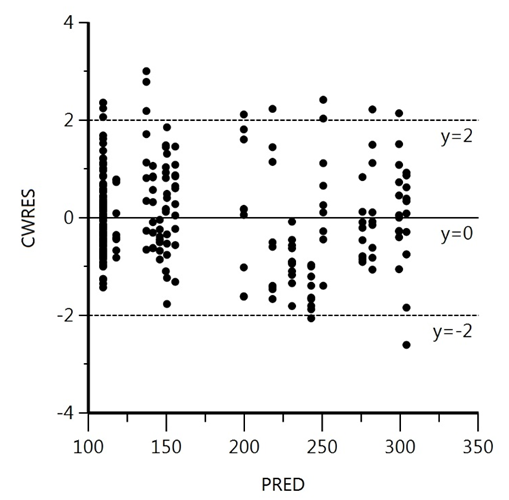 |

**Mouse PK/PD modelling**

The same structural model that was used for the analysis of the human and rat plasma TAG data was used for the analysis of the mouse data. Inter-individual variability was estimated on ka_TAG_, Tlag_TAG_, IC_50_ and R0 parameters according to a log-normal distribution of individual parameters. Inclusion/Exclusion of inter-individual variability in the different parameters was guided by quality of the model output and evaluation of the objective function. Residual error was calculated for plasma TAG using a proportional error model.

Effects of AZD7687 in the plasma TAG time course after SMM was well described by the PK/PD model proposed. The estimated PK/PD parameters are shown in Table 6. Diagnostic plots of the goodness of the fit are shown in Fig 10.

**Table 6:** Mouse PK/PD parameter Estimates for effects of AZD7687 in Plasma TAG following OLTT. IIV is Inter-Individual variability.

| Parameter | Value | Units | %CV |
| --- | --- | --- | --- |
| tvTlag_TAG_ | 0.98 | hr | 0.5 |
| tvKa_TAG_ | 2.10 | 1/hr | 15.8 |
| tvV_TAG_ | 0.012 | dL | 28.6 |
| tvR0 | 178.07 | mg/dL | 4.2 |
| tvkout | 4.41 | 1/hr | 23.4 |
| tvIC_50_ | 0.08 | umol/L | 33.1 |
| IIV lag_TAG_ | 0.19 | % | 31.2 |
| IIV Ka_TAG_ | 35.3 | % | 48.7 |
| IIV R0 | 17.7 | % | 41.5 |
| IIV IC_50_ | 62.0 | % | 71.3 |
| TAG Residual Multiplicative | 0.198 |  | 7.7 |

**Figure 10:** Goodness of fit plots for plasma TAG in mouse. A) Observed data (DV) versus individual predicted (IPRED) values. B) Observed data (DV) versus population predicted (PRED) values C) Conditional Weighted Residuals (CWRES) versus time (IVAR). D) Conditional Weighted Residuals (CWRES) versus predicted values (PRED)

| A  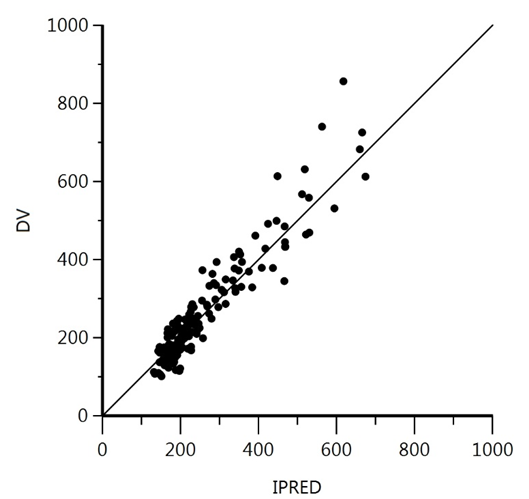 | B  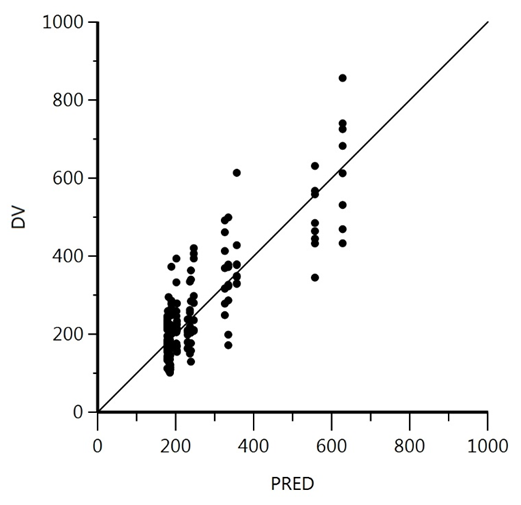 |
| --- | --- |
| C  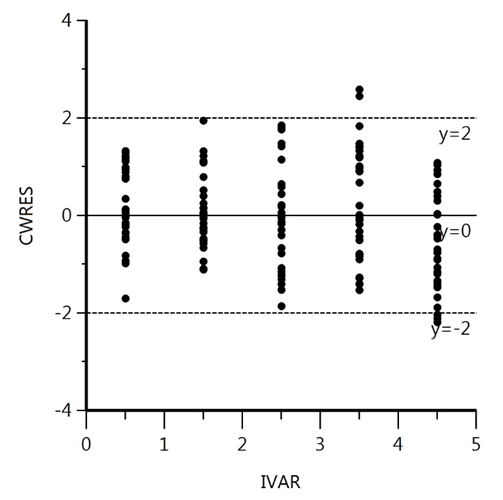 | D  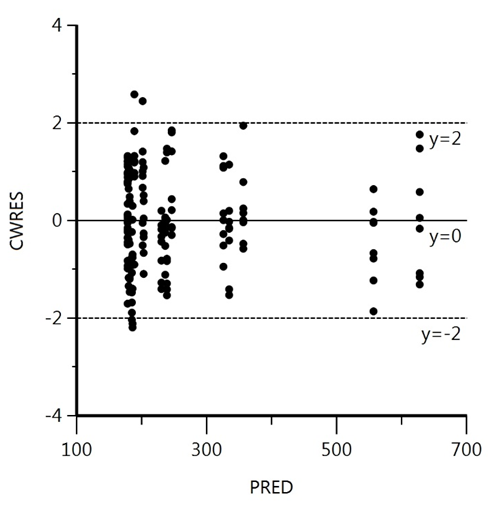 |
